# CurrentView: a tool for visualization and comparison of nanopore ionic current signals

**DOI:** 10.1093/bioinformatics/btag161

**Published:** 2026-04-10

**Authors:** Pooria Daneshvar Kakhaki, Neda Ghohabi Esfahani, Stuart Akeson, Miten Jain

**Affiliations:** Department of Electrical & Computer Engineering, Northeastern University, 360 Huntington Ave, Boston, MA 02115, United States; Department of Bioengineering, Northeastern University, 360 Huntington Ave, Boston, MA 02115, United States; Department of Bioengineering, Northeastern University, 360 Huntington Ave, Boston, MA 02115, United States; Department of Bioengineering, Northeastern University, 360 Huntington Ave, Boston, MA 02115, United States; Department of Physics, Northeastern University, 360 Huntington Ave, Boston, MA 02115, United States; Khoury College of Computer Sciences, Northeastern University, 360 Huntington Ave, Boston, MA 02115, United States

## Abstract

**Summary:**

Nanopore sequencing measures ionic current as native DNA or RNA molecules move through a biological pore. The resulting ionic current changes are inferred into sequence by Oxford Nanopore Technologies’ Dorado basecaller. These data permit direct analysis of nucleotide sequences and modifications. The Dorado basecaller also outputs a move-table that contains approximate mappings between ionic current signal and basecalled sequence. This ionic current information can be visualized at specific positions given the alignment between a read sequence and a reference sequence. We present CurrentView, a fast and user-friendly toolkit for reference-guided visualization of nanopore ionic current signals. CurrentView uses conventional sequence alignment and move-table information from BAM files and signal information from ONT POD5 files to extract and visualize ionic current traces at specific positions. The toolkit supports simultaneous comparison across multiple experimental conditions, computes summary statistics through kernel density estimation and histograms, and enables visual analysis of signal patterns associated with modifications or sequence context. Notably, CurrentView can visualize and analyze more than two conditions at once. It supports UMAP dimensionality reduction and Gaussian Mixture Model (GMM) clustering, enabling the identification of distinct signal populations across experimental groups. CurrentView is available as both a Python API and an exploratory interactive web application, allowing researchers to rapidly inspect ionic current patterns and compare conditions.

**Availability and implementation:**

CurrentView is fully open-source and available on GitHub https://github.com/genometechlab/currentview. The repository includes full documentation, an installation guide, usage instructions, and an example Jupyter notebook showing typical use cases similar to the one presented in the manuscript.

## 1 Introduction

Nanopore sequencing enables direct, real-time analysis of nucleic acids by measuring ionic current signals as individual RNA or DNA molecules translocate through a biological pore (Workman *et al.* 2019, Jain *et al.* 2022). Deep learning-based tools, or basecallers, can infer nucleotide sequences from these ionic current signatures (Akeson *et al.* 2025). Unlike sequencing-by-synthesis technologies, nanopore sequencing reads native molecules and is uniquely suited for directly detecting chemical modifications of nucleotides (Garalde *et al.* 2018). Oxford Nanopore Technologies (ONT) recently updated their DNA and RNA sequencing platforms (R10 for DNA and RNA004 for RNA). The combination of the Dorado basecaller and updated sequencing chemistry has improved DNA and RNA sequencing throughput, accuracy, and *de novo* detection of nucleotide modifications (Esfahani *et al.* 2025, Tzadikario *et al.* 2025).

Since multiple nucleotides (“k-mer”) within the pore contribute to the ionic current, assigning signal regions to individual bases is challenging (Laszlo *et al.* 2014, Wang *et al.* 2021). To address this, k-mer-based pore models (typically 5-mer or 9-mer) are used to approximate the expected ionic current mean and variance for a given nucleotide combination (Samarakoon *et al.* 2025). These models have been central to traditional signal alignment tools like Nanopolish eventalign (Leger *et al.* 2021, Hendra *et al.* 2022), F5C (Gamaarachchi *et al.* 2020) and Tombo. These software tools used k-mer models with dynamic programming algorithms to align ionic current signals to the expected reference k-mer through a process called resquiggling, or signal alignment (Gamaarachchi *et al.* 2020). This signal-to-reference alignment has been used for modification detection and downstream statistical analyses (Leger *et al.* 2021).

Dorado basecaller offers an alternative approach through movetable, a signal-to-read alignment that maps blocks of ionic current signal timepoints to basecalled nucleotides without requiring k-mer models. Each move-table entry indicates whether a base was emitted from a specific block of signal samples given the model’s stride interval. Stride is the number of data points per segment of ionic current that are used by the basecaller model for inferring nucleotide sequence. This coarse sequence-to-signal mapping can be output by Dorado using the “—emit-moves” flag and provides an approximate but direct link between ionic current and sequence. While basecallers and modification detection tools can achieve high accuracy using the ionic current and derived sequence information, direct inspection of the underlying signal remains essential for understanding model predictions, identifying novel modification signatures, and troubleshooting sequencing artifacts.

By leveraging the move-table for signal extraction, researchers can visualize ionic current without the computational overhead and potential inaccuracies of full signal-to-reference alignment. However, existing visualization tools are often slow, difficult to use, and typically limited to comparing only two conditions at once. Here we present CurrentView, a fast and user-friendly toolkit for the visualization of nanopore sequencing signals. CurrentView uses move-table from Dorado-based BAM files and raw signal data from POD5 files to extract and visualize ionic current traces at user-defined regions within a conventional alignment. This alignment can be achieved by using either minimap2 (map-ont parameter) or bwa mem (ont2d parameter). The toolkit supports comparison of multiple experimental conditions simultaneously with a flexible window size. This permits multi-sample signal comparison, position-wise summary statistics analysis through kernel density estimation, and Gaussian mixture model clustering to identify signal subpopulations. CurrentView is open-source and available via GitHub as both a Python package with a programmatic API and an interactive web application for exploratory analysis.

## 2 Materials and methods

CurrentView consists of four modules: (i) ionic current visualization for displaying raw signal traces at specific positions, (ii) summary statistical feature comparison for investigating position-wise signal distributions through kernel density estimation, (iii) UMAP embedding for low-dimensional representation of signal features, enabling visual separation of read populations across conditions, and (iv) Gaussian mixture model clustering for identifying signal subpopulations. For input, CurrentView requires aligned BAM files containing a move-table (and MD tags for base identity), along with the corresponding POD5 directory containing the ionic current signals.

### 2.1 Data preparation

For using CurrentView, the basecalls (BAM file) must be generated using the—emit-moves flag within Dorado. This option allows for the inclusion of move-table tags (mv, ts, pi, sp, ns) that map the signal timepoints to basecalled nucleotides. During the sequence alignment, tags must be preserved. This can be done by retaining the tags during data processing. This requires: (i) using samtools with the -T “*” flag when extracting FASTQ reads from the BAM file and (ii) using the -y flag during the minimap2 alignment step. It is important to note that a transcriptome reference needs to be used for alignment, along with the map-ont parameter for minimap2. If using bwa-mem for alignment, the -C flag is required. The aligned BAM file should include MD tags for assigning base identity (using samtools calmd). Lastly, the BAM file must be sorted and indexed.

### 2.2 Ionic current visualization

The ionic current visualization module displays raw nanopore signals aligned to reference positions. Users specify a k-mer window size (K), which defines the number of nucleotide positions to display centered on the position of interest. The module supports visualization of single or multiple experimental conditions, enabling direct comparison of signal patterns across samples (e.g. control versus treatment versus wild type). For each condition, users should specify the respective BAM file and POD5 folder paths, the target chromosome/contig, and the reference position of interest. The user can define the molecule type to either RNA or DNA. Optional parameters allow filtering of primary aligned reads, filtering by specific read IDs, limiting the maximum number of reads displayed or excluding reads containing insertions and deletions (exclude_reads_with_indels), and defining a perfect query match with reference position (matched_query_base). Users can apply custom normalization or transformation functions to the raw signal before visualization.

During visualization, each nucleotide is assigned a unit-length interval on the x-axis, spanning from *n* to *n* + 1, according to its reference position within the k-mer window. The raw ionic current samples corresponding to that nucleotide are then linearly distributed across this interval. Specifically, if a nucleotide is associated with k signal samples, these samples are plotted at *k* evenly spaced x-coordinates between *n* and *n* + 1. This approach preserves the original signal amplitudes while enabling variable-length nucleotide signals to be visualized within a common sequence-aligned coordinate framework.

Plot aesthetics are customizable (through the “PlotStyle” class), including line width, color, transparency, and style (solid, dashed, dotted). The transparency mode can be set to “auto” (adjusted based on read count) or “fixed” for consistent opacity. Plots can be saved in multiple formats (PNG, PDF, SVG) with adjustable resolution. The module also supports position highlighting and text annotations to mark features of interest within the k-mer window.

### 2.3 Summary statistics feature comparison

The statistical feature comparison module generates kernel density estimate (KDE) plots and histograms for position-wise signal statistics across the k-mer window. Users specify which summary statistics to calculate during initialization, including standard deviation, mean, median, signal duration (dwell time), maximum, minimum, skewness, and kurtosis. Additionally, users can pass custom functions to compute any signal-derived metric of interest.

For each position in the k-mer window, the module calculates statistics across the specified number of reads in a condition and plots their distributions as KDEs. When multiple conditions are visualized, overlaid KDE plots enable direct comparison of signal characteristics between samples. This facilitates identification of positions where signal properties differ significantly, which may indicate nucleotide modifications or sequence-dependent signal variations. The KDE plots share the same customization options as signal visualizations and can be displayed separately or alongside raw signal plots (using show_stats()).

### 2.4 UMAP dimensionality reduction

To visualize high-dimensional signal patterns across multiple reference positions, CurrentView employs Uniform Manifold Approximation and Projection (UMAP) for dimensionality reduction. UMAP characterizes each read using signal statistics computed across a window of positions flanking the target site. Users can specify which statistics to include (from both built-in metrics and user-defined functions) and also define an offset window. For each read, the selected statistics are computed at each position within the window and concatenated into a high-dimensional feature vector.

UMAP then reduces these multi-position feature vectors to a 2D embedding space by fitting a single model on the combined data from all conditions. This shared embedding ensures that reads from different conditions are projected into a common coordinate system, enabling direct visual comparison of their distributions. The resulting visualization displays each condition as a scatter plot in the reduced space, where proximity between points reflects similarity in their underlying signal patterns across the positional window.

This approach is particularly useful for identifying condition-specific clusters, detecting batch effects, or revealing complex ionic current signatures that span multiple positions. The embeddings provide an intuitive overview of global structure in the data, allowing users to explore high-dimensional signal patterns in an accessible 2D visualization without making parametric assumptions about the underlying distributions.

### 2.5 Gaussian mixture models

To investigate signal deviations that may correspond to nucleotide alterations or artifacts, CurrentView uses Gaussian mixture model (GMM) clustering. This module characterizes each read using signal statistics computed across a window of positions specified by the user. Users can select two statistics (including user-defined statistics as well as built-in metrics such as median signal level, signal duration, standard deviation, and mean) to define a 2D feature space for clustering. The GMM fits a specified number of components (e.g. 2 for modified versus unmodified or control versus wild type) to the combined data from each condition, treating each read as a data point in the bivariate feature space. After fitting, the 2D representation of each read is assigned to a cluster, and the module visualizes the distribution of reads across clusters for each condition. This approach can reveal subpopulations within the data that may represent distinct sequence contexts.

The module provides visualizations of cluster assignments and cluster centroids (means), allowing users to inspect whether identified clusters correspond to biologically relevant signal patterns or artifacts. This module also enables users to perform pairwise Kolmogorov–Smirnov tests and to compute Jensen–Shannon distance/divergence for comparing fitted Gaussian mixture models and their associated populations.

### 2.6 Runtime and memory usage

The runtime and memory usage of the tool are dependent on the size of the input BAM and POD5 directories (specifically the total number of reads contained within them), the number of reads the user requests to visualize, and the user’s hardware specifications. The tool employs pysam and the POD5 Python library to parse and prepare the respective inputs, meaning that larger datasets and a higher number of fetched reads will require proportionally greater processing time and memory allocation.

As a reference, in our experiments using a BAM and POD5 directory each containing 2000 reads, extracting a total of 400 reads required ∼4.6 s, while rendering the full visualization, including both signals and statistics took 13.9 s. These benchmarks are for an Apple MacBook Air equipped with an Apple M1 chip, 8-core CPU, 8-core GPU, and 16 GB of unified memory.

## 3 Usage example

To demonstrate CurrentView, we used Nanopore data for two synthetic RNA oligonucleotides from previous work (Esfahani *et al.* 2025), with and without a pseudouridine (Ψ) modification at a known position. These data were basecalled using Dorado v.1.0 and preprocessed following the steps described in the data preparation section. Based on prior work (Esfahani *et al.* 2025), Dorado called the known Ψ position as modified with 97.59% occupancy and the corresponding IVT canonical position as unmodified with 99.85% confidence (Esfahani *et al.* 2025).

**Figure 1:**
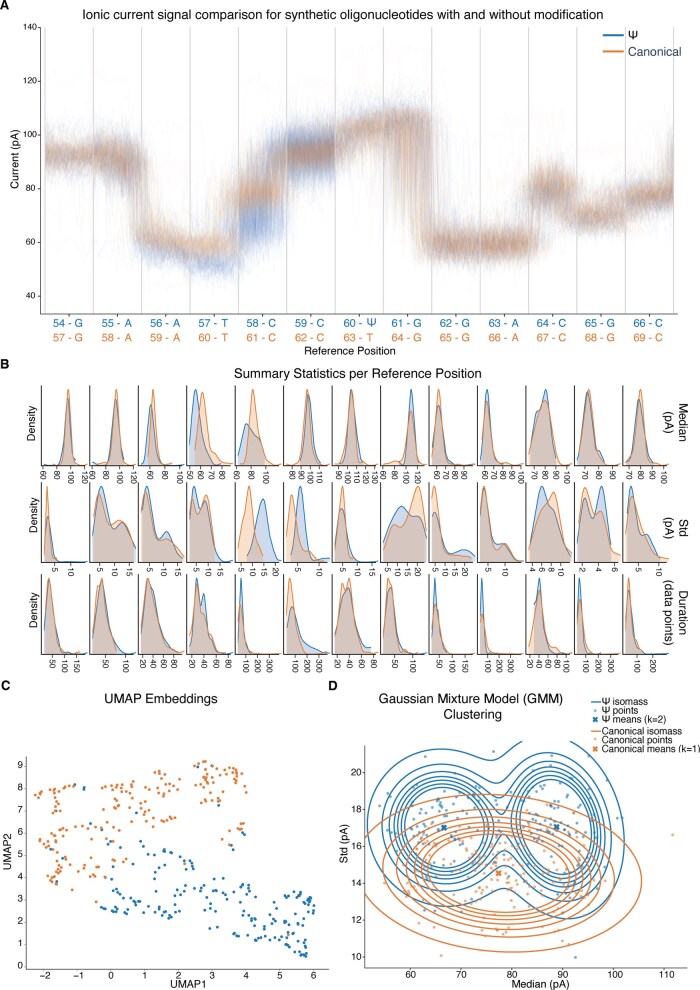
Visualization of pseudouridine modification in synthetic oligonucleotides using CurrentView. (A) Ionic current signal comparison between Ψ-containing oligonucleotide (blue) and canonical IVT oligonucleotide (orange) across a 13-base window. Each trace represents a single read (*n* = 100 per condition). Vertical gray lines indicate k-mer position boundaries, with reference position coordinates and bases labeled at the bottom. (B) Position-wise kernel density estimates for median signal intensity, standard deviation (Std), and dwell time (Duration). (C) UMAP embeddings based on aggregated median signal intensity and signal standard deviation computed across a 5mer window (positions 56–60 and 59–63). For each read, a feature vector is constructed by concatenating the mentioned features for each of the positions in the window. This window was selected through manual inspection of signal and statistics plots, targeting the region where the conditions exhibited the greatest divergence in signal characteristics. (D) Gaussian mixture model clustering based on median signal intensity and signal standard deviation computed across a 3-base window (positions −4 to −1 relative to the central position of each condition). The Ψ-containing sample (blue) exhibits two distinct clusters, while the canonical sample (orange) shows a single population. Contour lines represent density isopleths, points represent individual reads, and X markers indicate cluster centroids.

We visualized the ionic current signal, summary statistics, and GMM clustering for both conditions using a 13-base window (*K* = 13) centered on the target position (T/Ψ), with 100 reads per condition (Fig. 1). The raw signal traces revealed deviation between the Ψ-containing oligonucleotide and the canonical IVT pair (canonical T base), with differences emerging at positions upstream of the Ψ site (Fig. 1A). Summary statistics comparison using kernel density estimation showed distinct distributions in median signal intensity, standard deviation, and dwell time at neighboring positions preceding the Ψ (Fig. 1B). UMAP embeddings based on aggregated median signal intensity and signal standard deviation showed two clear populations (Fig. 1C). GMM clustering identified two subpopulations in the Ψ-containing sample and a single population in the canonical sample, consistent with the presence of modification, with a Jensen–Shannon divergence of 0.3845 (Fig. 1D).

It is important to note that not all modifications are expected to produce a visually identifiable signal difference, as some of them can generate subtle signatures. While these patterns could require complex analysis methods, they still benefit from a visual inspection and primary analysis. To that end, CurrentView enables rapid exploration of regions of interest and comparison across multiple conditions in a fast, user-friendly manner, facilitating hypothesis generation and quality control in modification detection workflows.

## 4 Discussion and conclusion

Direct inspection of ionic current signals is helpful for understanding basecaller behavior, modification detection, nucleotide prediction validation, and identifying novel signal patterns. While deep learning models can capture complex features in raw signals, visual exploration remains critical for interpreting model outputs and investigating unexpected results. CurrentView addresses the need for fast, accessible signal visualization by providing a user-friendly interface for exploring ionic current signals from RNA and DNA at specific reference positions with various filtering criteria. The toolkit supports simultaneous visualization of multiple conditions, enabling more comprehensive analyses and comparisons across diverse experiments.

CurrentView offers flexibility in data selection and filtering. Custom read IDs can be specified to analyze selected subsets of reads, while reads containing insertions or deletions can be excluded, and filtering can be applied to retain only reads with positional reference matches. Position-wise summary statistics and Gaussian mixture modeling can be utilized to model characteristic signal differences between two conditions. These capabilities enable analyses to be tailored to specific experimental questions and quality control requirements.

By bridging the visualization gap between ionic current signal and corresponding sequence, CurrentView facilitates quality control, interpretation of nanopore data, and hypothesis generation. The combination of speed, ease of usage, flexibility, and multi-condition support makes CurrentView a practical tool for exploratory analysis.

## Data Availability

Example data are available on GitHub (https://github.com/genometechlab/currentview/tree/main/example).

## Software and code availability

CurrentView is fully open source (with MIT license) and available on GitHub (https://github.com/genometechlab/currentview). It requires no specialized hardware or software dependencies beyond standard Python libraries and runs on standard computers. Signal extraction and visualization are computationally lightweight, typically completing within seconds to minutes, depending on the number of reads. The repository includes full documentation, an installation guide, usage instructions, and an example Jupyter notebook showing typical use cases similar to the one presented in the manuscript. The repository contains example datasets used in this study. A user-friendly interactive web application is accessible at GitHub and compatible with standard systems. A tutorial for how to use the web application is provided in the repository.
